# Effect of Hemodialysis on Plasma Glucose Profile and Plasma Level of Liraglutide in Patients with Type 2 Diabetes Mellitus and End-Stage Renal Disease: A Pilot Study

**DOI:** 10.1371/journal.pone.0113468

**Published:** 2014-12-19

**Authors:** Takeshi Osonoi, Miyoko Saito, Atsuko Tamasawa, Hidenori Ishida, Daisuke Tsujino, Rimei Nishimura, Kazunori Utsunomiya

**Affiliations:** 1 Nakakinen Clinic, Department of Internal Medicine, Naka, Ibaraki, Japan; 2 Division of Diabetes, Metabolism and Endocrinology, Department of Internal Medicine, Jikei University School of Medicine, Minato-ku, Tokyo, Japan; 3 Graduate School of Public Health, University of Pittsburgh, Pittsburgh, Pennsylvania, United States of America; University of Tolima, Colombia

## Abstract

The effect of hemodialysis on the plasma glucose profile and liraglutide level after liraglutide injection was investigated in patients with diabetes and end-stage renal disease (ESRD). Either 0.6 mg or 0.9 mg liraglutide was subcutaneously administered daily to 10 Japanese type 2 diabetic patients with ESRD. Hemodialysis was conducted on days 1 and 3. Plasma liraglutide and glucose concentrations were measured by enzyme-linked immunosorbent assay and a continuous glucose monitoring system, respectively. The safety profile of liraglutide was also assessed. Hemodialysis had no effect on the pharmacokinetic parameters of liraglutide in patients with diabetes and ESRD; the maximum plasma concentration (C_max_), t_max_, area under the concentration-time curve (AUC), and CL/f were unaltered. Similarly, hemodialysis did not affect the mean or minimum glucose levels, AUC, or duration of hyperglycemia (>180 mg/dL) and hypoglycemia (<70 mg/dL) following liraglutide administration. However, significant increases in mean amplitude of glycemic excursions (MAGE) and standard deviation (SD) as markers of glucose fluctuation, and the maximum glucose level were observed during hemodialysis. No adverse events, including hypoglycemia, were observed after liraglutide injection, either off-hemodialysis (day 2) or on-hemodialysis (day 3). Liraglutide was well tolerated in patients with type 2 diabetes and ESRD undergoing hemodialysis. The present results suggested that hemodialysis did not affect the pharmacokinetic profile of liraglutide or most glycemic indices, with the exception of MAGE, SD, and the maximum glucose level. These results suggested that it may be possible to use liraglutide during hemodialysis for diabetes with ESRD, without dose adjustment.

***Trial Registration*** UMIN Clinical Trials Registry (UMIN-CTR) UMIN000010159

## Introduction

Type 2 diabetes is a major risk factor for chronic renal diseases and many affected individuals develop diabetic nephropathy, the leading cause of end-stage renal disease (ESRD). In Japan, approximately 95% of diabetic patients with ESRD receive hemodialysis [1]. The therapeutic options for diabetic patients with ESRD requiring hemodialysis are generally limited due to the accumulation of anti-diabetic drugs in the body by the reduction of glomerular filtration rate (GFR) and leading to hypoglycemia [2].

The efficacy and safety of liraglutide, a long-acting glucagon-like peptide (GLP-1) receptor agonist, have been investigated in type 2 diabetic patients [3]–[5]. However, evaluation of the efficacy and safety of liraglutide in non-diabetic and diabetic patients with severe renal impairment is limited to several small studies [6], [7]. The effect of renal impairment on the pharmacokinetics of liraglutide in 24 Caucasian patients with renal impairment was reported previously [6]. This study evaluated the pharmacokinetics of liraglutide (0.75 mg, single-dose subcutaneous injection) in non-diabetic patients with varying degrees of renal impairment. Compared to healthy subjects, patients with mild, moderate, or severe renal impairment and ESRD showed, no increase in plasma exposure of liraglutide, indicating that the pharmacokinetics of liraglutide were not influenced by renal function. These results in non-diabetic patients suggested that liraglutide dose adjustment might not be required in diabetic patients with ESRD. The efficacy and safety of low-dose liraglutide (0.3 mg) have recently been reported in nine Japanese diabetic patients with ESRD undergoing hemodialysis, using a continuous glucose monitoring system (CGMS) [7]. The results of this study suggested that liraglutide had good efficacy and was well-tolerated in type 2 diabetic patients with ESRD switching from insulin therapy to liraglutide and requiring hemodialysis. However, no previous reports have described the measurement of plasma liraglutide concentrations in diabetic patients with ESRD undergoing hemodialysis. Therefore, it is important to investigate whether the liraglutide dose adjustment is required in diabetic patients requiring hemodialysis, by evaluating its pharmacokinetic profile, efficacy and safety of liraglutide, due to the high prevalence of renal dysfunction and limited to therapeutic options of anti-diabetic drugs in the patients. Our primary objective was to assess the efficacy of liraglutide in controlling blood glucose levels and to evaluate its effect on hypoglycemia in these patients. Our secondary objective was to evaluate the effect of hemodialysis on the plasma levels of liraglutide in type 2 diabetes patients with ESRD.

## Materials and Methods

The protocol for this trial and supporting TREND checklist are available as supporting information; see Checklist S1 and Protocol S1.

### Ethics and Good Clinical Practice

This study was conducted in accordance with the International Conference on Harmonisation (ICH) Good Clinical Practice (GCP) guidelines [8] and Ethical Guidelines for Clinical Studies in Japan [9]. The investigators complied with all applicable regulatory and legal requirements, ICH GCP guidelines, and the Declaration of Helsinki [10] in obtaining and documenting informed consent. Subject confidentiality was strictly maintained and no subject was involved in any trial-related activity without obtaining appropriate written informed consent. The study protocol was reviewed by the ethics committee at Nakakinen Clinic. Ethics committee approval was obtained before the initiation of this study.

### Patients

This single-center, open-label, pilot study was conducted in 10 diabetic patients with type 2 diabetes and ESRD (UMIN Clinical Trial Registry 000010159) at Nakakinen Clinic. The period of patient recruitment was between January 28, 2013 and February 28, 2013. The specific patient eligibility criteria are listed below. Inclusion criteria: male and female patients with type 2 diabetes and ESRD; age ≧20 years; treated with a stable dose of 0.6 mg or 0.9 mg liraglutide for at least 2 weeks; glycoalbumin (GA) ≤30%; able to provide informed consent before any trial-related activities. Patients were excluded if they had type 1 diabetes; had been treated with another incretin mimetic within 3 months prior to screening; had impaired hepatic function (measured as serum glutamic oxaloacetic transaminase [also known as aspartate aminotransferase, AST] or serum glutamic pyruvic transaminase [also known as alanine aminotransferase, ALT])>100 IU/L; had unstable cardiovascular or cerebrovascular disease; were pregnant or breast-feeding; intended to become pregnant or were female and of childbearing age and not using adequate contraceptive methods; had known or suspected allergy to trial medication(s), excipients, or related products; and had any contraindications to liraglutide.

### Study Design

A group of 10 patients with type 2 diabetes and ESRD requiring hemodialysis received liraglutide (subcutaneous administration) at a dose of either 0.6 or 0.9 mg (Victoza; Novo Nordisk A/S, Denmark) from the investigators at Nakakinen Clinic, using the Victoza pen device. The 0.9 mg dose of liraglutide is the highest approved clinical dosage in Japan. Nine patients received 0.9 mg liraglutide and 1 patient received 0.6 mg. Patients were admitted to hospital on day 1 and underwent hemodialysis (hemodialyzers: NV-16U, NV-10U, and NV-21U [Toray Medical Co. Ltd., Chiba, Japan], FX 140 and FX 180 [Fresenius Medical Care, Germany], APS-18SA [Asahi Kasei Medical Co. Ltd., Tokyo, Japan]; blood flow rate of 160–220 mL/min; dialysate flow rate of 450 mL/min; replacement flow rate of 0 mL/min; filtration flow rate of 1.3–3 L/h). On day 2, the patients with CGMS received a dose of liraglutide at 06∶00 hours and blood samples (2 mL) were collected at the following time points: 06∶00 hours (pre-dose), 08∶00, 12∶00, 15∶00, 18∶00, 21∶00, and 06∶00 (day 3). These time points equated to 0, 2, 6, 9, 12, 15, and 24 h post-dose. Hemodialysis was not conducted in these patients on day 2. On day 3, patients with CGMS received a further dose of liraglutide at 06∶00 hours and blood samples were collected at the same time points as on day 2. In addition, patients underwent hemodialysis between 08∶00 and 12∶00 hours and an additional arterial blood sample (2 mL) was collected at the end of the hemodialysis session. On day 4, blood samples were collected at 06∶00 hours (as 24 hour-samples on day 3).

### Efficacy Assessments

Plasma liraglutide concentrations were measured by a validated liraglutide-specific enzyme-linked immunosorbent assay (ELISA) method at Huntingdon Life Sciences (Cambridgeshire, UK). Pharmacokinetic parameters were calculated from the plasma liraglutide concentrations at each sampling point off-hemodialysis (day 2) and on-hemodialysis (day 3) using the WinNonlin software. The area under the concentration-time curve (AUC_0-τ_), maximum plasma concentration (C_max_), t_max_, CL/f, and C_dialysate_ were calculated.

Plasma glucose concentration was measured using CGM System-Gold (Medtronic MiniMed, Northridge, CA, USA) since CGM is useful for evaluation of the effect of liraglutide on glucose level fluctuation in diabetic patients [11]. CGM was performed both off-hemodialysis (day 2) and on-hemodialysis (day 3). The maximum and minimum glucose levels, average of 24-h glucose levels, standard deviation (SD), and mean amplitude of glycemic excursions (MAGE) of 24-h glucose, AUC, and the duration of hyperglycemia (>180 mg/dL) and hypoglycemia (<70 mg/dL) on-hemodialysis (day 3) and off-hemodialysis (day 2) were compared using SAS version 9.3 and Microsoft Excel 2003.

### Safety Assessments

The severity and causal relationship to liraglutide of all treatment-related adverse events in these patients were assessed by the investigators. A physical examination, including vital signs (blood pressure and heart rate) and clinical laboratory biochemical tests, was also performed on each patient.

### Statistical Methods

Analyses of the plasma pharmacokinetic and glucose profiles were performed at Huntingdon Life Sciences and the New Drug Research Center, Inc. (Tokyo, Japan), respectively.

The mean, maximum, and minimum glucose concentrations; SD; and MAGE were analyzed using a paired t-test and 95% confidence interval (CI) for the mean ratio (on-hemodialysis/off-hemodialysis). Because these values could be logarithmically transformed, the transformed data were used for analyses. Some values for duration and AUC of hyperglycemia (>180 mg/dL) and hypoglycemia (<70 mg/dL) could not be transformed logarithmically because zero values were included. In these cases, the original non-transformed values were analyzed by paired t-test. Similarly, the plasma glucose and pharmacokinetic parameters (C_max_, AUC_0-τ_, and CL/f) were analyzed using 95% CI for the mean on-hemodialysis/off-hemodialysis ratio. Values of p<0.05 were considered significant.

## Results

### Patient Demographics and Baseline Characteristics

**Figure 1:**
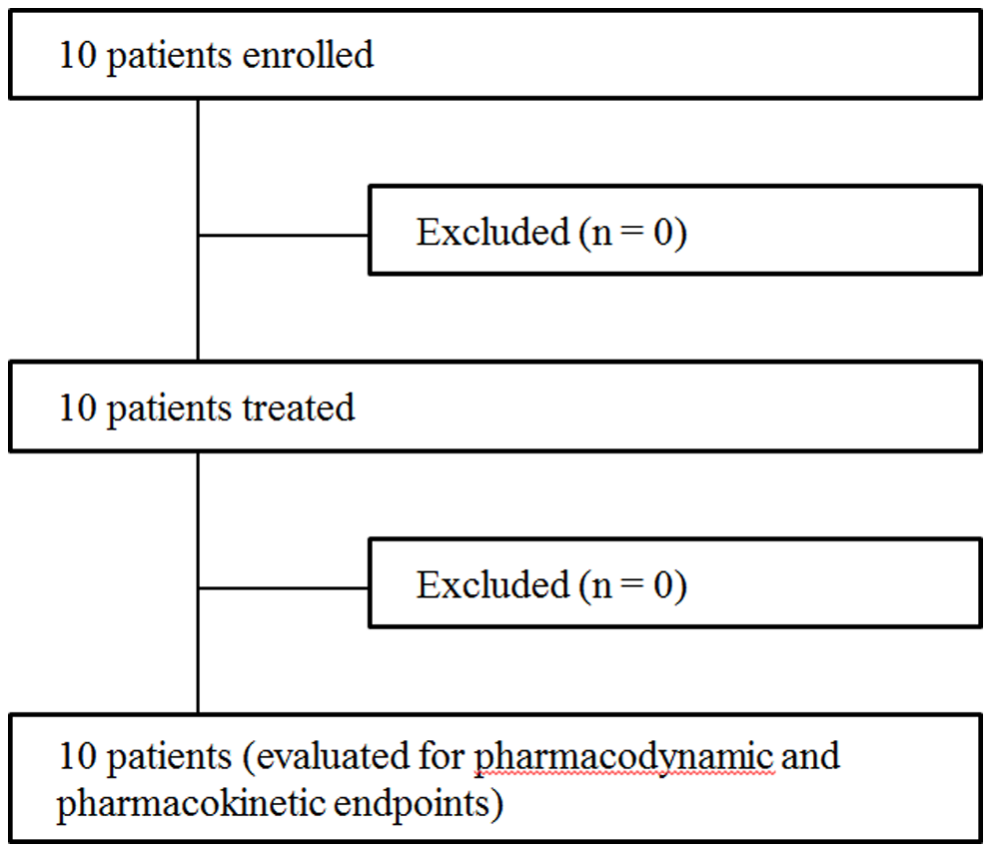
Patient disposition.

All 10 patients completed the study, and these patients were included in the pharmacodynamic and pharmacokinetic evaluations (Fig. 1). The baseline demographics and characteristics of all of these Japanese patients are shown in Table 1.

**Table 1:** Baseline demographics and characteristics of the patients.

Characteristic	Values*
Age (years)	65.8±9.3
Body weight (kg)	57.9±9.8
BMI (kg/m^2^)	21.4±2.6
Sex male/female**	9/1
Duration of diabetes (years)	20.1±9.0
HbA_1c_ (%)	5.9±0.9
GA (%)	21.0±2.8
Creatinine (mg/dL)	11.4±2.5
BUN (mg/dL)	62.4±16.9
eGFR (mg/min/1.73 m^2^)	4.2±1.3
Total cholesterol (mg/dL)	152.7±38.7
Triglyceride (mg/dL)	125.5±68.8
AST (U/L)	11.2±4.5
ALT (U/L)	10.2±6.0
CPK (IU/L)	74.6±30.5
Uric acid (mg/dL)	7.5±0.9
Pre-treatment history	
Liraglutide only (n)	9
Insulin and liraglutide*** (n)	1

*Values are mean ± SD (n = 10).

**0.9 mg (8 males and 1 female), 0.6 mg (1 male).

***1 patient had previously been treated with insulin only, and had switched to liraglutide 3 months prior to initiation of the study.

AST: Aspartate aminotransferase (SGOT).

ALT: Alanine aminotransferase (SGPT).

BUN: Blood urea nitrogen.

CPK: Creatinine phosphokinase.

Participants included 9 male patients and 1 female patient with an age of 65.8±9.3 (mean ± SD) years, body weight of 57.9±9.8 kg, and body mass index (BMI) of 21.4±2.6 kg/m^2^. The duration of diabetes was 20.1±9.0 years. The baseline HbA_1c_ and GA were 5.9±0.9% and 21.0±2.8%, respectively. The diabetic patients with ESRD in this trial had high levels of creatinine (11.4±2.5 mg/dL) and blood urea nitrogen (BUN, 62.4±16.9 mg/dL), and a reduced estimated GFR (eGFR, 4.2±1.3 mL/min/1.73 m^2^). The total cholesterol, triglyceride, AST, ALT and creatinine phosphokinase (CPK) values were within their normal reference ranges, but uric acid (7.5±0.9 mg/dL) was slightly higher than the normal reference range. With regard to pre-treatment history, 9 patients had received liraglutide only more than 2 years prior to the initiation of the study. In addition, 1 patient had previously been treated with insulin only, and had switched to liraglutide 3 months prior to initiation of the study.

### Efficacy

The time-change profile of the plasma liraglutide concentration and 95% CI for the mean ratios at each time point on-hemodialysis (day 3) and off-hemodialysis (day 2) are shown in Table 2. The 95% CI for each sampling time included 1. The t_max_ on-hemodialysis (day 3) and off-hemodialysis (day 2) was generally 10.5 or 9.5 h after liraglutide injection, respectively. Hemodialysis did not appear to have any effect on t_max_ values. Pharmacokinetic parameter values (AUC_0-τ_, C_max_, t_max_, and CL/f) and 95% CI for their mean ratios are shown in Table 3. The mean pharmacokinetic parameter ratios (on-hemodialysis/off-hemodialysis) were generally close to 1 and all 95% CIs included 1, indicating that hemodialysis had little or no effect on C_max_, AUC_0-τ_, or CL/f after subcutaneous administration of liraglutide. The ratio of the venous to the arterial plasma liraglutide concentrations was 0.92, close to 1, indicating that arterial and venous plasma concentrations of liraglutide at the end of the hemodialysis session were similar. Plasma liraglutide concentrations in the pooled dialysate samples were below the limit of quantitation (<30 pM) for all patients, indicating that little or no liraglutide was able to cross the dialysis membrane.

**Table 2:** Time-change profile of plasma liraglutide concentration following subcutaneous administration of liraglutide in diabetic patients with ESRD on the days of on-hemodialysis and off-hemodialysis.

Type of Hemodialysis	Off-hemodialysis (day 2)	On-hemodialysis (day 3)	Mean ratio
	p mol (Mean±SE)	p mol (Mean±SE)	(95% Confidence interval)
2 hr	15479±4413	19224±5280	1.25 (0.96–1.62)
6 hr	18663±5218	21185±6188	1.12 (0.86–1.45)
9 hr	19568±4930	21582±6260	1.08 (0.83–1.40)
12 hr	19392±5053	20483±5289	1.06 (0.82–1.38)
15 hr	18441±5206	20939±6674	1.10 (0.84–1.42)
24 hr	14486±4902	16670±6107	1.14 (0.88–1.49)

n = 10.

**Table 3:** Pharmacokinetic parameters of liraglutide following subcutaneous administration of liraglutide to diabetic patients with ESRD on the day of on-hemodialysis and off-hemodialysis.

PK parameters	Off-hemodialysis (day 2)	On-hemodialysis (day 3)	Mean ratio
	Mean ±SD	Mean±SD	(95% Confidence interval)
AUC_0-τ_ (p mol)	415700±373200	473100±455900	1.11 (0.97–1.26)
C_max_ (p mol)	20940±16130	22620±20590	1.02 (0.86–1.22)
t_max_ (hours)	9.5±3.3	10.5±2.5	-
CL/f (mL/min)	200±125	188±151	0.90 (0.79–1.03)

-: not calculated, n = 10.

The effects of hemodialysis on plasma glucose concentrations and markers of glucose fluctuation after the administration of liraglutide are shown in Table 4. A significant increase in SD, MAGE, and maximum glucose level was observed on-hemodialysis, as compared with off-hemodialysis. However, hemodialysis on day 3 did not significantly affect the mean and minimum glucose levels, or the duration and AUC of hyperglycemia and hypoglycemia, as compared with those observed off-hemodialysis (day 2). No sign or symptom of hypoglycemia was observed after administration of liraglutide off-hemodialysis (day 2) or on-hemodialysis (day 3), but 2 patients showed a slight reduction in plasma glucose level (<70 mg/dL) for a short period on both days (off-hemodialysis [day 2]: to 67 mg/dL for 10–15 min in 2 patients; on-hemodialysis [day 3]: to 61 mg/dL for 55 min in 1 patient and to 68 mg/dL for 5 min in another patient).

**Table 4:** Plasma glucose profiles after subcutaneous administration of liraglutide in diabetic patients with ESRD on-hemodialysis and off-hemodialysis.

Measurement items	Off-hemodialysis(day 2)	On-hemodialysis(day 3)	Mean ratio(95% Confidence interval)	t-testp values
Mean glucose level (mg/dL)	**133.0±36.5**	138.7±35.3	1.05 (0.98–1.12)	0.158
Maximum glucose level (mg/dL)	**188.4±62.4**	226.2±100.9	1.16 (1.02–1.32)	0.026**
Minimum glucose level (mg/dL)	**92.7±27.1**	88.4±21.4	0.96 (0.82–1.13)	0.596
SD (mg/dL)	**22.1±12.4**	30.5±20.1	1.32 (1.04–1.67)	0.028**
MAGE (mg/dL)	**55.6±27.4**	90.0±65.7	1.44 (1.05–1.98)	0.027**
Time (min) during 24 hr inhyperglycemia (>180 mg/dL)	**214.5±388.4**	210.0±282.9		0.928
AUC (mg/dL•min) during 24 hr inhyperglycemia (>180 mg/dL)	**7746.0±17246.4**	11427.5±19184.4		0.127
Time (min) during 24 hr inhypoglycemia (<70 mg/dL)	**2.5±5.4**	6.0±17.3		0.575
AUC (mg/dL•min) during 24 hr inhypoglycemia (<70 mg/dL)	**5.0±10.5**	36.5±112.0		0.406

Mean ± SD, n = 10.

*The analysis of 95% CI (log [CI]) could not be performed because zero values were included.

**p<0.05.

### Safety

No adverse events, including signs of hypoglycemia, were observed in diabetic patients with ESRD after the administration of liraglutide off-hemodialysis (day 2) or on-hemodialysis (day 3). No clinical changes in the physical examination or vital signs (blood pressure and heart rate) were observed. The values of eGFR, total cholesterol, triglyceride, AST, ALT and CPK on day 3 after admission were very similar to those recorded before admission. However, BUN, creatinine and uric acid levels were significantly improved because of hemodialysis in these patients (BUN: 62.4±16.9 to 26.5±8.3 mg/dL [p<0.01]; creatinine: 11.4±2.5 to 5.9±1.4 mg/dL [p<0.01]; uric acid: 7.5±0.9 to 3.1±0.7 mg/dL [p<0.01]).

## Discussion

This single-center, open-label, pilot study was conducted in 10 Japanese patients with type 2 diabetes and ESRD requiring hemodialysis in order to investigate the effect of hemodialysis on the plasma glucose profile, plasma levels of liraglutide, and safety following subcutaneous administration of liraglutide. The present results suggested the possibility that a dose adjustment of liraglutide is not required in diabetic patients with ESRD requiring hemodialysis because hemodialysis did not affect the plasma glucose profile (mean and minimum glucose levels, or duration and AUC of hyperglycemia and hypoglycemia), the pharmacokinetics, or the safety of liraglutide. It has already been reported that MAGE and SD were significantly higher on-hemodialysis than off-hemodialysis in type 2 diabetic patients treated with insulin therapy [12]. The present study also found increases in SD and MAGE, with significant differences between on-hemodialysis and off-hemodialysis.

The therapeutic anti-diabetic drug options are limited for diabetic patients with ESRD requiring dialysis because the associated reduction in GFR causes accumulation of unchanged drugs and active metabolites [2], leading to the induction of hypoglycemia. Therefore, conventional anti-diabetic drugs such as sulfonylureas are not suitable because of the risk of prolonged hypoglycemia in these patients [13]; recently approved DPP-4 inhibitors are used (with an appropriate dose reduction) in diabetes patients with severe renal impairment [14]. A GLP-1 receptor agonist, exenatide, is not recommended for use in patients with ESRD because its AUC_0–∞_ and C_max_ after in non-diabetic patients with ESRD were 3.37 and 1.38 times higher than those observed in healthy controls, respectively [15].

Insulin treatment remains the main therapy for the control of hyperglycemia in diabetic patients with ESRD requiring hemodialysis, but it sometimes induces severe hypoglycemia. Liraglutide binds plasma proteins, preventing rapid elimination from the circulation [16], and is therefore considered suitable for diabetic patients with ESRD in which GFR is reduced due to renal impairment. In the present study, since there were no significant differences in pharmacokinetic parameters calculated on-hemodialysis and off-hemodialysis after subcutaneous administration of liraglutide, hemodialysis was not found to affect the pharmacokinetics of liraglutide in these patients. The reason why the liraglutide concentration was not affected by hemodialysis was unclear. It is possible that the high level of liraglutide binding to albumin and α-acidic glycoprotein (approximately 99%) prevented liraglutide from passing through the dialysis membrane, resulting in no decrease in plasma liraglutide levels during hemodialysis.

In addition, different degrees of renal dysfunction were not found to increase exposure of liraglutide in a previous clinical pharmacology study [6]. Taken together with the findings of the present study, this indicated that liraglutide dose adjustment was not required in patients (diabetic or non-diabetic) with renal impairment. However, there is extremely limited experience with liraglutide in diabetic patients with ESRD undergoing hemodialysis and careful monitoring after liraglutide administration to these patients is therefore required.

The efficacy and safety of liraglutide (at a low dose of 0.3 mg) in diabetic patients with ESRD undergoing hemodialysis was recently reported by Terawaki et al. [7]. In that study, the mean period of hypoglycemia after liraglutide administration was approximately 50 min/day and a higher frequency of hypoglycemia was observed after liraglutide injection than after administration of insulin or DPP-4 inhibitors such as vildagliptin and alogliptin.

In the present study, no signs or symptoms of hypoglycemia were observed in diabetic patients with ESRD off-hemodialysis (day 2) or on-hemodialysis (day 3) after the administration of higher liraglutide doses of 0.6 mg (n = 1) and 0.9 mg (n = 9). However, when hypoglycemia was defined as a glucose level <70 mg/dL in the present study, the duration of hypoglycemia in 2 patients during the off-hemodialysis period was 10–15 min, when their glucose levels were mildly reduced (to 67 mg/dL), whereas the duration of hypoglycemia in 2 patients on-hemodialysis was 55 min in 1 patient (61 mg/dL) and 5 min in another patient (68 mg/dL). One study reported that liraglutide did not cause hypoglycemia because it stimulated insulin secretion in a glucose-dependent manner [17], which may explain why the frequency of hypoglycemia after liraglutide injection in diabetic patients with ESRD was lower than that observed using insulin products, which easily induce hypoglycemia in diabetic patients with renal impairment. However, studies with larger sample sizes and longer treatment periods are required to further investigate the level of hypoglycemia after liraglutide administration in diabetic patients with ESRD requiring hemodialysis. In addition, hemodialysis did not affect the AUC or duration of hyperglycemia (>180 mg/dL) in the present study.

No adverse events were observed in 10 diabetic Japanese patients with ESRD after the administration of liraglutide (0.6 mg/day or 0.9 mg/day) during the off-hemodialysis or on-hemodialysis days in the present study. A previous study of liraglutide (0.3 mg/day) in nine diabetic Japanese patients with ESRD also reported no adverse events [7]. A separate study reported some adverse events (2 events of vomiting and 1 event of nausea) after a single injection of liraglutide (0.75 mg) in non-diabetic Caucasian patients with varying degrees of renal impairment [6]; the degree of renal impairment of the patients in this study was not associated with an increased risk of adverse events. Vomiting and nausea are the most common adverse events observed when first starting liraglutide injection in diabetic patients, but these tend to decrease with continued liraglutide treatment [18]. It is likely that the present study observed no vomiting or nausea because the patients had been treated with a stable dose of 0.6 mg or 0.9 mg liraglutide for at least 2 weeks before the trial commenced. The safety results of the present and previous studies suggested that liraglutide was well tolerated and could be used without dose adjustment during hemodialysis in diabetic patients with ESRD.

## Conclusions

Liraglutide was well-tolerated in patients with diabetes and ESRD undergoing hemodialysis and off-hemodialysis. Hemodialysis did not increase the risk for adverse clinical effects or altered pharmacokinetic profiles after liraglutide injection in these patients. Therefore, these results suggested that liraglutide may be used without dose adjustment during hemodialysis in patients with diabetes and ESRD.

## Supporting Information

Checklist S1 **TREND Statement Checklist.**
(PDF)

Protocol S1 **Trial Protocol.**
(PDF)
