# Supplementary material for: Effect of Hemodialysis on Plasma Glucose Profile and Plasma Level of Liraglutide in Patients with Type 2 Diabetes Mellitus and End-Stage Renal Disease: A Pilot Study
Source: PLoS One. 2014 Dec 19;9(12):e113468. doi: 10.1371/journal.pone.0113468 (PMC4272272; doi:10.1371/journal.pone.0113468)
Supplement: Protocol S1 — Trial Protocol. (PDF) [file pone.0113468.s002.pdf]

Effect of Haemodialysis on the Plasma glucose profile and Plasma level of Liraglutide in People with Type 2 Diabetes Mellitus and End Stage Renal Disease

INVESTIGATOR-INITIATED STUDY PROPOSAL

UNIVERSAL TRIAL NUMBER (UTN)：U1111-1137-0366

Takeshi Osonoi, MD
Director
Nakakinen Clinic
Ibaraki, Japan

Kazunori Utsunomiya, MD
Professor of Internal Medicine
Director
Division of Diabetes, Metabolism & Endocrinology
Jikei University School of Medicine

Correspondence to:

Kazunori Utsunomiya, MD
Professor of Internal Medicine
Director
Division of Diabetes, Metabolism & Endocrinology
Jikei University School of Medicine

List of Abbreviations

ADR	Adverse drug reaction
AE	Adverse event
ASAT	Aspartate Aminotransferase
AUC	Area under the curve
ESRD	End Stage Renal Disease
DM	Diabetes Mellitus
HbA1c	Glycosylated haemoglobin 1c
ICH	International Conference on Harmonisation
GCP	Good Clinical Practice
SAE	Serious Adverse Event
SADR	Serious Adverse Drug Reaction
sGOT	Serum glutamic oxaloacetic transaminase
sGPT	Serum glutamic pyruvic transaminase

BACKGROUND AND SIGNIFICANCE:

The effect of renal impairment on the pharmacokinetics of liraglutide has been studied previously[1-]. A study has been conducted to evaluate the single-dose pharmacokinetics of liraglutide in patients with varying degrees of renal impairment. Subjects with mild (estimated creatinine clearance 50-80 mL/min) to severe (estimated creatinine clearance <30 mL/min) renal impairment and subjects with end-stage renal disease requiring dialysis were included in the trial. Compared to healthy subjects, liraglutide area-under the curve (AUC) in mild, moderate, and severe renal impairment and in end-stage renal disease was on average 33%, 14%, 27% and 26% lower, respectively. It is important that liraglutide be used carefully in these subjects.

In Japan, approximately, 95% of patients with End Stage Renal Disease (ESRD) undergo haemodialysis. We have recently examined the efficacy and safety of liraglutide in type 2 Diabetes Mellitus (DM) patients with ESRD who were treated with haemodialysis[2-]. In that study, 19 non-insulin-requiring patients were switched to liraglutide from existing treatment with insulin. After one month of follow-up, there was no acute impairment of blood glucose level in any of the subjects. Glycosylated haemoglobin 1c (HbA1c) showed slight decrease from 5.3±0.5 to 5.1±0.5 (p= 0.07). Glycoalbumin (GA) after one-month treatment of liraglutide improved from 21.0±2.2 to 19.4±2.4% (p= 0.02). Prior to switching to liraglutide, 30% of the subjects complained of hypoglycemia symptoms. After the switch, hypoglycemia symptoms were not observed. Feeling of hunger decreased in 40% of patients. Two patients were withdrawn from liraglutide treatment due to nausea, and 3 of them needed treatment with prokinetic agents. Our experience indicate that in patient with type 2 diabetes undergoing hemodialysis, liraglutide induction is considered to be useful in terms of safety and efficacy.

In our small previous study, we have not measured the effect of haemodialysis on the plasma glucose profile measured by Continuous Glucose Monitoring (CGM) and plasma levels or clearance of liraglutide, and we are not aware of any published reports on these specific parameters. It is important to provide stronger scientific and safety rationale for the use of liraglutide in type 2 DM patients with ESRD treated with hemodialysis. The purpose of this current proposal is to investigate the effect of hemodialysis on the plasma liraglutide level in these subjects.

OBJECTIVES:

Our primary objective will be to assess the efficacy of liraglutide in controlling blood glucose levels and evaluate its effect on hypoglycemia in these patients. Our secondary objective will be to evaluate the effect of haemodialysis on the plasma levels of liraglutide in type 2 diabetes patients with ESRD.

STUDY AIMU AND HYPOTHESIS

Study Hypothesis (hypotheses):

We hypothesize that liraglutide will be safe and effective in treating subjects with type 2 DM and ESRD.

Primary Aim: to measure the change in plasma glucose profile measured byCGM on-haemodialysis and off- haemodialysis.

PrimaryHypothesis: Use of liraglutide on and off haemodialysis will not significantly affect the periods of hypoglycaemia measured by CGM.

Secondary aim: to compare the plasma levels of liraglutide before and after haemodialysis in subjects who have been receiving a stable dose of liraglutide for 2 weeks after the initial titration period.

Secondary Hypothesis: liraglutide plasma level will not be significantly different before and after haemodialysis.

Endpoints:

Primary endpoint:
Plasma glucose profile (CGM) on-haemodialysis and off-haemodialysis

Secondary endpoint(s):
Plasma liragutide levels while on-haemodialysis and off-haemodialysis

Study type and description:

This will be a single centre, open-label study. We will enrol 10 subjects with type 2 DM and ESRD requiring haemodialysis. Subjects should be receiving stable dose of liraglutide and are in-stable glycemic control as evidenced by GA 30% or less.

The study will be conducted in two sampling periods with schedule shown in section "Visit Procedures". Subjects with Type 2 Diabetes and ESRD who are undergoing haemodialysis will be administered stable dose of liraglutide of either 0.6 mg or 0.9 mg for a minimum of 2 weeks after the titration period. Subjects will be instructed to administer liraglutide in the morning during this study. We will collect a set of 7 plasma samples to measure liraglutide levels in the 10 subjects during the haemodialysis procedure, then we will collect another set of 7 samples from the same subjects while they are not undergoing haemodialysis.

Administration of liraglutide and plasma sampling will be conducted at following time points:

On Day 1, The subjects will be admitted to the hospital to undergo haemodialysis.Subject will be treated haemodialysis in morning (08:30 – 13:00) of Day 1 and set CGM for calibration during haemodialysis.

On Day 2, subjects with CGM will be administered liraglutide on standardized time (06:00). Blood sample will be taken at 7 time-points at 0 (Just before dosing at 06:00, S1), 08:00 (S2), 12:00 (S3), 15:00 (S4), 18:00(S5), 21:00 (S6), and at 06:00 on Day 3 (Just before administration of liraglutide, S7) without being treated with haemodialysis. Blood Sampling should be conducted within ±1 hour from nominal sampling time because of practical feasibility at the site but sampling at S1 must be conducted before the administration of liraglutide.

On Day 3, subjects with CGM will be administered liraglutide at the same time and dose and taken blood samples at same time points as in Day 2 but treated with haemodialysis from 08:00 till 12:00 (4 hours) according to standardized timeline in the hospital. At the sampling point of S3, arterial blood sample will be taken in order to investigate the impact of haemodialysis on clearance of liraglutide. Dialysate will be pooled to measure liraglutide in dialysate. Blood Sampling should be conducted within ±1 hour from nominal sampling points because of practical feasibility at the site but sampling at S1 must be conducted before the administration of liraglutide.

On Day 4, blood samples will be taken on 06:00 (Just before administration, S7 in Day 3). After the administration of liragltuitide, Subjects will be examined and will leave from hospital.

Plasma will be treated with EDTA-3K according to standardized methods at the site. Liraglutide levels in plasma samples will be measured with the liraglutide-specific Enzyme-Linked ImmunoSorbent Assay (ELISA) method-by Huntingdon life science.

CGM will be monitored by supplier.

Pharmacokinetic parameters will be calculated by using WinNolin software in Huntingdon life science for: $AUC_{0-\tau}$, t_{max}, C_{max}, $t_{1/2}$, CL/f, $C_{dialysate}$ for plasma samples taken on-haemodialysis and off-haemodialysis.

Study population:

Ten subjects with type 2 DM and ESRD will be studied. The specific eligibility criteria are listed below:

Inclusion Criteria

1- Male and female subjects with type 2 Diabetes Mellitus
2- Age ≥20 years
3- Treated with stable dose of liraglutide of 0.6mg or 0.9mg for at least 2 weeks.
4- GA 30% or less
5- Able to provide informed consent before any trial-related activities.

Exclusion Criteria

1- Type 1 Diabetes Mellitus
2- Previous treatment with other incretin mimetics within the previous three months before screening.
3- Insulin-requiring type 2 Diabetes Mellitus
4- Impaired hepatic function, measured as serum glutamic oxaloacetic transaminase [sGOT (Aspartate Aminotransferase , ASAT)] or serum glutamic pyruvic transaminase (sGPT) ≥ 100 IU/L
5- Unstable cardiovascular or cerebrovascular disease.
6- Pregnant, breast-feeding or the intention of becoming pregnant or not using adequate contraceptive measures.
7- Female of childbearing potential who are not using adequate contraceptive menthods
8- Known or suspected allergy to trial medication(s), excipients, or related products.
9- Any contraindications to liraglutide.

Withdrawal Criteria

1- The subject may withdraw at will at any time.
2- Pregnancy or intention of becoming pregnant
3- At the investigator's discretion to ensure subject's safety.

Subject Replacement

Withdrawn subjects will be replaced on a 1:1 basis to have 10 subjects completing both on-haemodialysis and off-haemodialysis. The data of withdrawn subjects will be excluded from analysis of primary and secondary endpoints. Data for all subjects will be included in the evaluation of safety.

Rationale for Study Population

In order to investigate primary objective, the subjects with type 2 diabetes and ESRD receiving the steady dose of liraglutide and achieving good blood glucose control are considered to be the most appropriate population to examine this objective. The results may be generalizable to a larger population with similar characteristics, which may provide additional important details in terms of the effectiveness and safety of liraglutide in this group of people.

Visit Procedures

Screening: Investigate adequacy of the subjects for inclusion and exclusion criteria for this study and informed consent to the subjects.

Day 1: Admission of the subjects to the hospital to undergo haemodialysis. Perform physical examination and assess the vital signs and other important parameters as part of routine examination. Subject will be treated haemodialysis in morning (08:30 – 13:00) of Day 1 and set CGM for calibration during haemodialysis.

Day 2: Assure the appropriate dose of liraglutide, CGM, Blood sampling to determine plasma liraglutide levels, Blood sampling for Clinical Laboratory AE. Perform physical examination and check vital signs.

Day 3, Administer liraglutide, CGM, Haemodialysis, Blood sampling to determine plasma liraglutide levels, Blood sampling for Clinical Laboratory AE. Perform physical examination and check vital signs.
In Day 4, Administration of liraglutide. Perform physical examination and check vital signs.

We have included below a Diagram showing study visits and timelines.

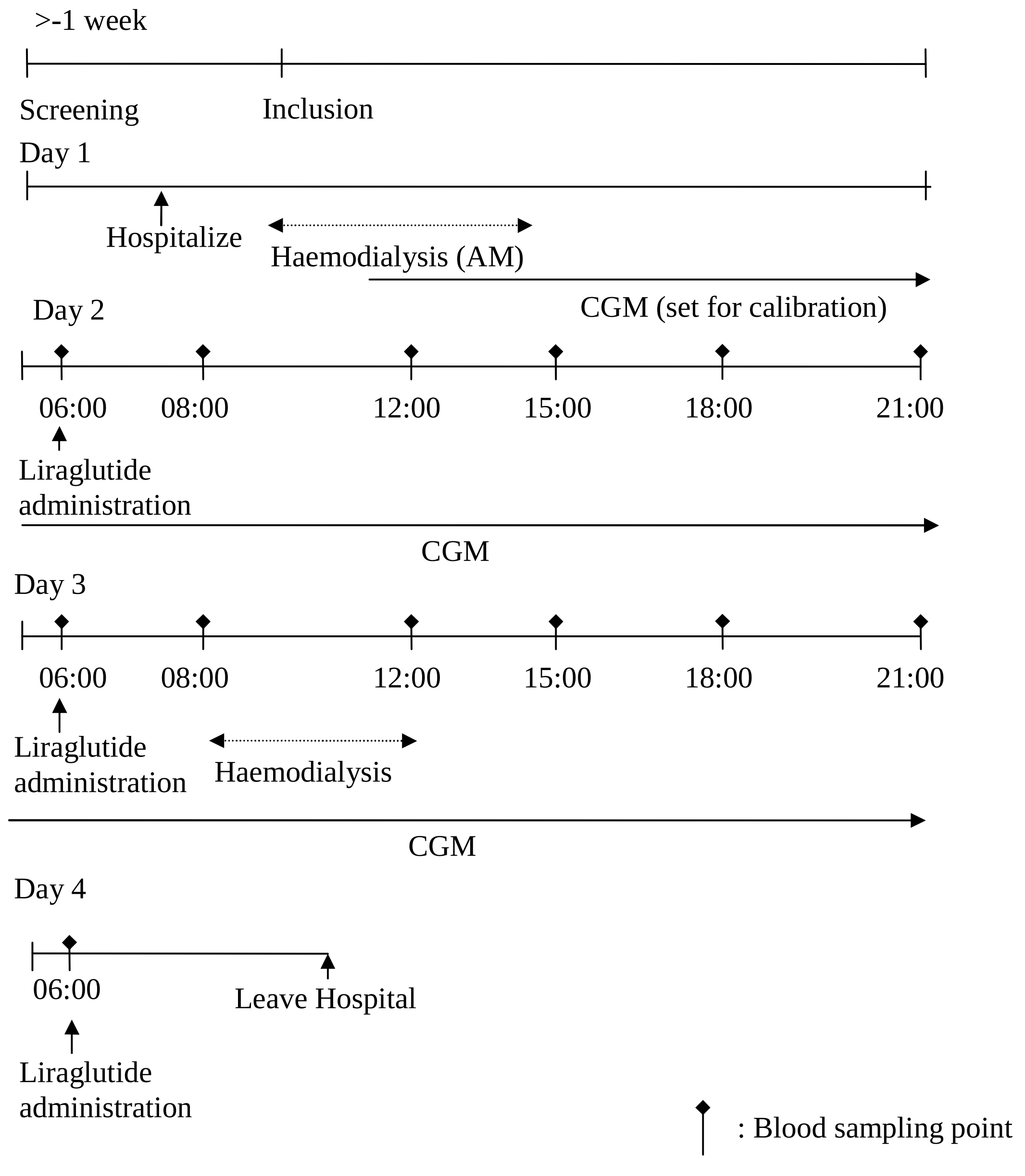

Assessments for Efficacy
Plasma glucose level based on CGM, Plasma liraglutide levels, Pharmacokinetic parameters

Assessments for Safety
Physical examination, Vital signs, Adverse events, Clinical laboratory tests (haematology, biochemistry and urinalysis)

STATISTICAL CONSIDERATIONS:

Sample Size Calculation
N=10
This sample size is assumed to be sufficient to meet the primary objective of this study, however, formal statistical calculation of sample size has not yet been performed.

Statistical Methods
Individual profile of the plasma glucose profile based on CGM by type of haemodialysis (on- or off-haemodialysis) will be graphically presented.

Individual profile of the plasma liraglutide profile as well as mean profile by type of haemodialysis (on- or off-haemodialysis) will be graphically presented. A mixed effect model will be fitted to the plasma liraglutide profile data. The model will include type of haemodialysis, time, interaction between type of haemodialysis and time as fixed factors and subject as a random effect. From the model, mean plasma liraglutide profile by type of haemodialysis and relevant differences will be estimated and explored.

Each pharmacokinetic (PK) parameter will be summarized descriptively by type of haemodialysis. If appropriate, each PK parameter will be analyzed using paired t- test. Mean ratio or mean difference between type of haemodialysis will be estimated and presented with corresponding 95% confidence interval.

DATA HANDLING AND RECORD KEEPING:

Nakakinen clinic is responsible for data management. Data management may be delegated under an agreement of transfer of responsibilities to other data management service unit within or outside Huntingdon life science (Data concerning to the determination of liraglutide in plasma and calculation of Pharmacokinetic parameters) or external Contract Research Organization [CRO (Laboratory data etc)] if needed and as appropriate.

The subject and the biological samples obtained during this study will be identified by subject code. Pharmacoknetic data will be identified with dummy subject identifier before data base lock. Appropriate measures such as encryption or deletion will be enforced to protect the identity of human subjects in all presentations and publications as required by local/regional/national requirements.

For the screening failures, the data of the screening failure form and affirmation statement will be entered into the database.
For the withdrawals, all data collected up to the date and time of withdrawal will be entered into the database.

ETHICS:

This study will be conducted in accordance with the International Conference on Harmonisation（ICH） Good Clinical Practice (GCP) guidelines[3-] and Ethical Guidelines for Clinical Studies in Japan[4-]. The investigators will comply with all applicable regulatory and legal requirements, ICH GCP guidelines and the Declaration of Helsinki[5-] in obtaining and documenting the informed consent. Subject confidentiality will be strictly maintained and no subject will be involved in any trial-related activity without obtaining appropriate written informed consent. The study protocol will be reviewed by ethics committee at Nakakinen Clinic. Ethics committee approval will be obtain prior to the initiation of this study.

STUDY SCHEDULE:

Recruitment period: 2 weeks
IRB in Nakakinen clinic: Jan 25 2013
Start of study: Jan 28 2013
FPFV: Jan 29 2013
LPLV: Feb. 16 2013
Integrated final study report: June 2013

STUDY DRUGS AND MATERIALS:

Study medication(s) / devices(s)
Subjects in this study will receive Liraglutide 0.6 or 0.9 mg (Victoza – Novo Nordisk A/S). Liraglutide will be administered subcutaneously using the Victoza pen device according to the currently approved Japanese label. Liraglutide is used marketed product, not supplied by Novo Nordisk A/S.

CONCOMITANT ILLNESSES AND MEDICATIONS:

Definitions:
Concomitant illness: any illness that is present at the start of the trial *(i.e. at the first visit).*
Concomitant medication: any medication other than the trial product(s) that is taken during the trial, including the screening and run-in periods.
Details of all concomitant illnesses and medication must be recorded at trial entry (i.e. at the first visit). Any changes in concomitant medication must be recorded at each visit. If the change influences the subject’s eligibility to continue in the trial, the Sponsor will be informed.

The information collected for each concomitant medication includes, at a minimum, start date, stop date or continuing, and indication.
For each concomitant illness, date of onset, date of resolution or continuing, at a minimum, should be recorded.

SAFETY REPORTING:

The investigator will be responsible for reporting of all adverse events including serious adverse events (SAE), serious adverse drug reactions (SADRs) to Novo Nordisk and the competent authority based upon the Japanese regulations and local institutional policies.

The investigator will collect the following information at minimum for each of these events:

1. Study name
2. Subject identification (e.g. initials, sex, age)
3. Event (preferably a diagnosis)
4. Drug (e.g. Norditropin Simplex®)
5. Reporter identification (e.g. Name, or initials)

6) Causality
7) Outcome

Applicable definitions within this study:

Adverse Event (AE):
An AE is any undesirable medical event occurring to a subject in a clinical trial, whether or not related to the trial product(s). This includes events reported from the first trial related activity from the subject has signed the informed consent and until leaving hospital. The following should not be recorded as AEs, if recorded as medical history/concomitant illness on the database at screening:

- Pre-planned procedure, unless the condition for which the procedure was planned has worsened from the first trial related activity after the subject has signed the informed consent
- Pre-existing conditions found as a result of screening procedures

Clinical Laboratory Adverse Event:
A clinical laboratory AE is any clinical laboratory abnormality regarded as clinically significant i.e. an abnormality that suggests a disease and/or organ toxicity and is of a severity, which requires active management, (i.e. change of dose, discontinuation of trial product, more frequent follow-up or diagnostic investigation).

Serious Adverse Event (SAE):
A serious AE is an experience that at any dose results in any of the following:

- Death
- A life-threatening* experience

• In-subject hospitalisation or prolongation of existing hospitalization
• A persistent or significant disability/incapacity
• A congenital anomaly/birth defect
• Important medical events that may not result in death, be life-threatening*, or require hospitalisation may be considered an SAE when, based upon appropriate medical judgement, they may jeopardise the subject and may require medical or surgical intervention to prevent one of the outcomes listed in this definition

*The term life-threatening in the definition of SAE refers to an event in which the subject was at risk of death at the time of the event. It does not refer to an event which hypothetically might have caused death if it was more severe.

Serious Adverse Drug Reaction (SADR):
An adverse drug reaction (ADR) is an adverse event for which a causal relationship (Possible/Probable relation) between the study drug and the occurrence of the event is suspected. The ADR should be classified as **serious** if it meets one or more of the seriousness criteria.

Non-Serious Adverse Event:
A non-serious AE is any AE which does not fulfil the definition of an SAE.

Severity Assessment Definitions:
• Mild: Transient symptoms, no interference with the subject's daily activities
• Moderate: Marked symptoms, moderate interference with the subject's daily activities
• Severe: Considerable interference with the subject's daily activities, unacceptable

Relationship to study medication Assessment Definitions:
• Probable: Good reasons and sufficient documentation to assume a causal relationship
• Possible: A causal relationship is conceivable and cannot be dismissed
• Unlikely: The event is most likely related to an etiology other than the trial product

Outcome Categories and Definitions:
• Recovered: Fully recovered or by medical or surgical treatment the condition has returned to the level observed at the first trial related activity after the subject signed the informed consent
• Recovering: The condition is improving and the subject is expected to recover from the event. This term should only be used when the subject has completed the trial
• Recovered with sequelae: As a result of the AE, the subject suffered persistent and significant disability/incapacity (e.g. became blind, deaf, paralysed). Any AE recovered with sequelae should be rated as an SAE
• Not recovered: The condition of the subject has not improved and the symptoms are unchanged, or the outcome is not known at the time of reporting
• Fatal: This term is only applicable if the subject died from a condition related to the reported AE. Outcomes of other reported AEs in a subject before he/she died should be assessed as "recovered", "recovering", "recovered with sequelae" or "not recovered". An AE with fatal outcome must be reported as an SAE
• Unknown: This term is only applicable if the subject is lost to follow-up

Collection, Recording and Reporting of Adverse Events
All adverse events will be collected from the first study- related activity (from the signing of the informed consent) and in following contact with the study subject untilnext visitng to hospital after leaving. .
The investigator should copy NN when expediting SADRs or unexpected SADRs to Competent Authorities and should report all SADRs related to Novo Nordisk Product to Novo Nordisk. The submission to Novo Nordisk must however be within day 15 from the Investigator getting knowledge about a valid case no matter local timelines for reporting to the authorities.

Follow-up of Adverse Events
During and following a subject's participation in this study, the investigators and hospital will provide adequate medical care to the study subject for any study-related adverse events, including clinically significant laboratory values related to the study.

All adverse events classified as serious or severe or possibly/probably related to the study product will be followed until the subject has recovered and all queries have been resolved. For cases of chronic conditions follow-up until the outcome category is "recovered" is not required, as these cases can be closed with an outcome of "recovering" or "not recovered".
All other adverse events will be followed until the next visiting to hospital after leaving. ,.

Pregnancy
The investigator will report to Novo Nordisk any pregnancy occurring in trial subject during use liraglutide . Reporting of pregnancy by sponsor-investigator will occur within the same timelines described above for reporting of Adverse Events.

Pregnancy complications will be recorded as adverse event(s). If the infant has a congenital anomaly/birth defect this must be reported and followed up as a serious adverse event.

Precautions/Over-dosage
Subjects will be instructed on the possible adverse events related to the use of liraglutide or its over-dosage. In the event of overdose, subjects will be treated according to the current guidelines and standards of care.

LIABILITY :

During and following a subject's participation in study, the investigator and host institution will provide adequate medical care to the study subject for any study-related adverse events, including clinically significant laboratory values related to the study. This medical care for study subjects will be provided regardless of their insurance status. The investigator will be responsible for the conduct of the study and the investigator agrees to defend, indemnify, and hold harmless Novo Nordisk, any of its parent companies, affiliates, or subsidiaries, and their respective officers, directors, employees, agents,

representatives, distributors, salespersons, customers, licensees, and end-users from and against any claim, suit, demand, loss, damage, expense or liability imposed by any third party arising from or related to: (a) any breach of investigator's obligations; or (b) investigator’s negligent or grossly negligent use or willful misuse of the study drug, the results, or services derived therefrom.

PUBLICATION PLAN:

Investigator will make good faith effort to publish the results of this study in a peer reviewed scientific journal or otherwise disclose publicly the data or results of this study. Authorship will be defined according to Vancouver/ICMJE criteria in the publications. Novo Nordisk will receive a copy of any publications for commenting prior to submission.

The investigator will register the study with a publicly assessable database such as clinicaltrials.gov.

REFERENCES:

1- Jacobsen LV. et al., Effect of renal impairment on the pharmacokinetics of the GLP-1 analogue liraglutide., Br J Clin Pharmacol. 68(6):898-905, 2009
2- Ishida H et al, II-2-14Examination of the GLP-1 analogue formulation induction in patient of type 2 diabetis using hemodialysis - Changes in early stages after introduction of liraglutide from Insulin- 54th Annual meeting of Japan Diabetic society (abstract), 2011
3- ICH E6(R1) Guideline for Good Clinical Practice, 27, March 1997, Ministry of Health, Labour and Welfare,
4- Ethical Guidelines for Clinical Studies-Amended 31 July, 2008, Ministry of Health, Labour and Welfare, 2008 (Japanese only)
5- World Medical Association Declaration of Helsinki: Ethical principles for medical research involving human subjects - Last amended by the 59th WMA General Assembly, Seoul 2008. http://www.wma.net/e/policy/b3.htm. 2008.
